# Differentiating malignancy from liver parenchyma in Ex-Vivo OCT images using anomaly detection

**DOI:** 10.1038/s41598-026-54850-0

**Published:** 2026-06-10

**Authors:** Ulrich Krispel, Alexander Pamler, Caroline Girmen, Niels König, Robert Schmitt, Eva Eggeling, Sören Büsker, Oliver Beetz, Felix Oldhafer, Daniel Truhn, Florian W. R. Vondran, Iakovos Amygdalos

**Affiliations:** 1 Fraunhofer Austria Research GmbH, Graz, 8010 Austria; 2 Fraunhofer Institute for Production Technology IPT, Aachen, 52074 Germany; 3 Laboratory for Machine Tools and Production Engineering (WZL) of RWTH Aachen University, Aachen, 52074 Germany; 4 Institute for Pathology, University Hospital RWTH Aachen, Aachen, 52074 Germany; 5 Department of General, Visceral, Pediatric, and Transplantation Surgery, University Hospital RWTH Aachen, Aachen, 52074 Germany; 6 Department of Diagnostic and Interventional Radiology, University Hospital RWTH Aachen, Aachen, 52074 Germany

**Keywords:** Cancer, Computational biology and bioinformatics, Gastroenterology, Oncology

## Abstract

Primary liver cancer and colorectal liver metastases (CRLM) pose significant challenges, because of limited early diagnosis and the reliance on time-consuming frozen section analysis during surgery to confirm complete tumor resection (R0). This study investigates the potential of optical coherence tomography (OCT) combined with anomaly detection for differentiating hepatocellular carcinoma (HCC), intrahepatic cholangiocarcinoma (iCCA) and CRLM from normal liver parenchyma, ex-vivo. Our dataset comprises 173 OCT images sourced from 69 patients undergoing liver surgery. We leveraged pre-trained neural networks with frozen weights and statistical outlier modeling to train an anomaly detection model using only non-cancer parenchyma scans. Given the small-scale nature of the dataset and the presence of label uncertainty, a stratified cross-validation procedure was employed to robustly assess the model’s performance in accurately matching OCT scans with their corresponding histological diagnoses. This resulted in promising classification performance using a pre-trained Vision Transformer: sensitivity 80%, specificity 78%, accuracy 79%, and area under the receiving-operating-characteristic-curve (ROC-AUC) of 81%. While limited by a relatively small and noisy dataset, this study highlights the promising potential of OCT combined with anomaly detection for intraoperative liver cancer detection. This semi-supervised learning approach offers several advantages, including reduced training time and data requirements, as well as interpretable anomaly scores.

## Introduction

Primary liver cancer is the sixth most common cancer worldwide, accounting for 4% of all cancer cases and causing the second highest number of cancer deaths (8%)^1,2^ after lung and colorectal cancer. It comprises hepatocellular carcinoma (HCC, up to 85% of cases), intrahepatic cholangiocarcinoma (iCCA, up to 15%), and other, rarer malignancies^1,2^. Moreover, colorectal cancer is the third most common cancer type worldwide, with the majority of these patients developing liver metastases, which are the primary cause of mortality^2–4^. As such, colorectal liver metastases (CRLM) are the most commonly diagnosed malignancy in the liver. Curative liver resection with complete tumor removal (R0) is the best option for all of these pathologies. However, only 12%-40% of patients diagnosed with iCCA are eligible for surgical therapy and curative therapies such as surgical resection and liver transplantation are restricted to a minority of HCC patients with early disease^1,5–8^.

This underlines the importance of improving diagnostics to detect liver cancer earlier and to accurately determine resection radicality intraoperatively. Additionally, the intraoperative detection of peritoneal metastases is crucial to the treatment plan, as these carry a difficult prognosis and often change a curative situation into a palliative one^9,10^. Intraoperative tissue diagnostics rely on frozen section analysis, which is time-consuming, especially when multiple tissue samples are examined^11–13^. This leads to longer surgeries, particularly when frozen sections are positive, requiring further clearance of the resection margin. This, in turn, is associated with an increased risk of complications and costs^14,15^.

Therefore, a technology with the potential to quickly conduct intraoperative tissue diagnostics would be an attractive solution to this problem. A non-invasive imaging technology with the potential to address these challenges is optical coherence tomography (OCT). Based on low-coherence interferometry, OCT produces real-time, high-resolution cross-sectional images with a depth of 1-3 mm, with axial and lateral resolutions of 1-20 $\upmu \hbox {m}$, respectively^12,13,16,17^. However, apart from previous work by our group^12,13,17^ on colorectal liver metastases and iCCA, no other studies in the literature have combined OCT with machine learning (ML) to differentiate between healthy and malignant tissue in human liver specimens. Furthermore, specific ML algorithms offer distinct advantages. In the case of anomaly detection, training can rely solely on control images^18^ (such as non-malignant tissue), to differentiate anomalies in aberrant images (i.e., tumors). Even though anomaly detection has been previously applied to OCT images, this has never been done on human liver tissues.

This study investigates the ability of OCT combined with anomaly detection to differentiate liver malignancies from normal liver parenchyma ex vivo.

## Related work

### OCT

**Fig. 1:**
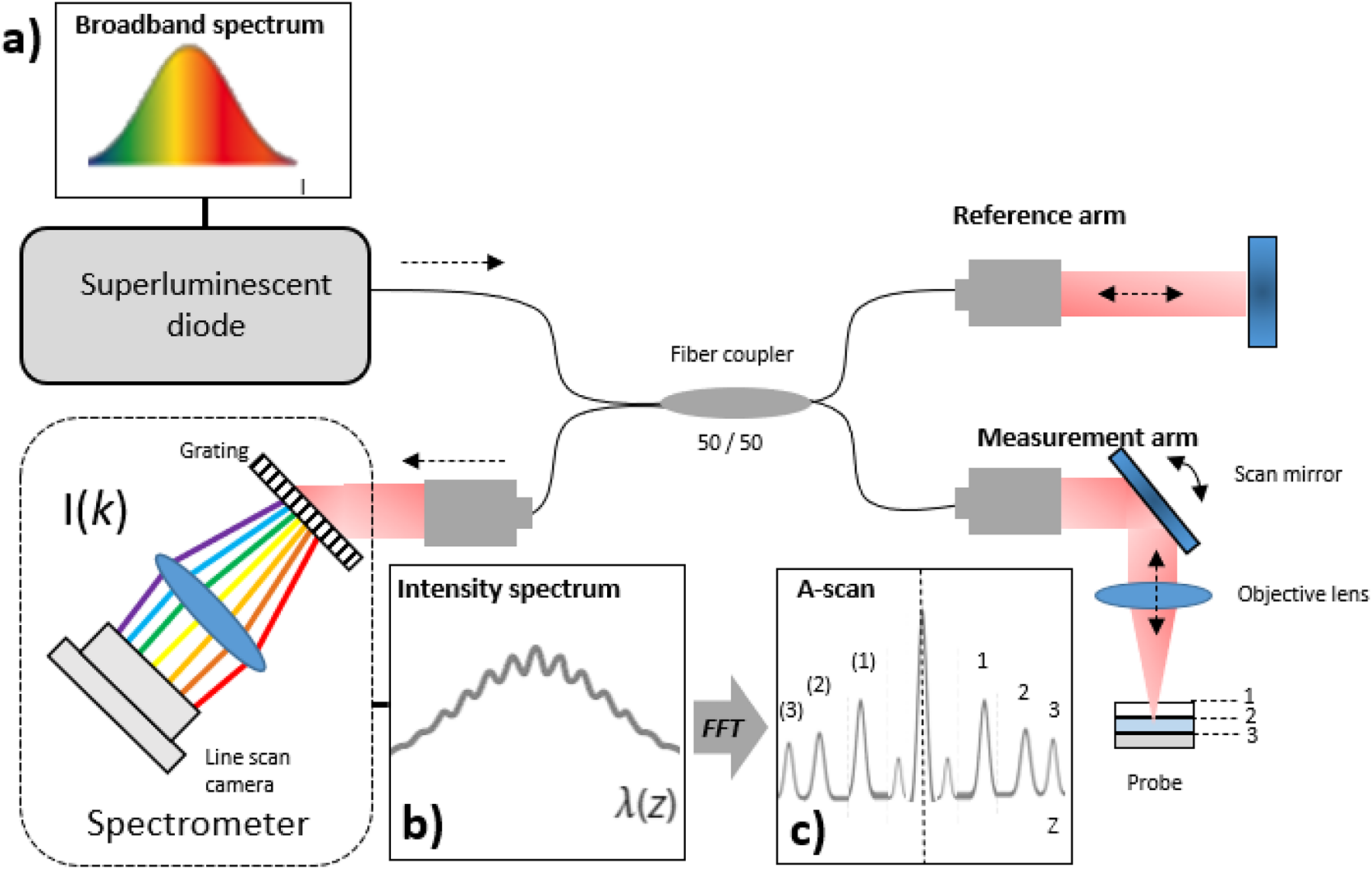
(**a**) Schematic setup of a Fourier domain OCT, (**b**) spectrogram at the detector unit and (**c**) back reflection profile with depths information.

Optical Coherence Tomography is a cross-sectional imaging technique first described in 1991 by Huang et al.^19^. It utilizes low-coherence interferometry to measure backscattered light’s echo time delay and magnitude. Three-dimensional cross-sectional scans can be acquired with spatial resolutions of 10 $\upmu \hbox {m}$ or less, penetrating millimeters into tissue. While the first OCT devices were developed as time-domain systems, used for imaging of the human retina in vitro first in 1993^20^, they improved in recent years, increasing in speed and resolution. The basic setup for Fourier domain OCT is shown in Figure 1, which involves a broadband light source split into two paths: one directed toward a reference mirror and the other toward the sample. Light is backscattered from the sample based on its optical properties. When recombined with the light reflected from the reference mirror, interference patterns are created and recorded by the detector as seen in the setup in Figure 1 b).

This pattern describes a one-dimensional A-Scan (Figure 1 c), capturing the backscattered intensity at various depths along the beam’s trajectory within the sample. 2D images can be composed of many A-scans by scanning the sample with the laser beam, referred to as B-scans. Multiple B-scans then form a volume scan (C-scan)^21^.

These setups usually result in an axial resolution between 2 and 15 $\mu$m with penetration depths of 0.5 - 3 mm for biological samples depending on the type of tissue as well as the wavelengths. With higher penetration depths, the signal-to-noise ratio decreases due to attenuation effects. Compared to standard ultrasound imaging, the resolution is about 100 times better, approaching the resolution of histopathology^22^. Besides that, the fiber-based setup, shown in Figure 1, offers the advantage of stable real-time measurements in various clinical environments with handheld probes such as endoscopes, catheters, or laparoscopes for non- or minimal invasive in-situ imaging. Currently, OCT is most frequently used in ophthalmology^23,24^ and dermatology^25–27^. As a result, OCT systems have been used to detect and diagnose cancer tissue in recent years^12,27–30^. Despite the comparatively high resolution of OCT systems, some characteristic features of cancerous tissue remain below their resolution limit. In addition, the resolution can be further limited by the lack of contrast in biological tissue^21^.

### Anomaly detection

The distinction between a healthy and non-healthy state is a common classification problem in biomedical engineering. This has led to the application of machine learning approaches, such as supervised, unsupervised, and semi-supervised settings, for building classifier models from labeled data.

Recent work has used OCT to differentiate colorectal liver metastases from liver parenchyma ex vivo^12,13^. This study employed supervised learning to classify between the two classes. However, supervised learning may suffer from data distribution shift, leading to overfitting on the training set and poor generalization to unseen samples. To mitigate this issue, we explore a semi-supervised approach in this work. This methodology is particularly interesting for the medical domain due to the scarcity of training samples^31^. Semi-supervised learning has also been applied in image-based anomaly detection tasks^32^, where models are trained on “good” examples and used to detect anomalies. Unsupervised approaches are actively researched in several medical domains, such as MRI^33^ and histopathology^34^.

While semi-supervised approaches such as autoencoders^35^ or GANs^36^ have shown promise in identifying anomalies in OCT images, they still require the computational overhead of training a deep neural network. In contrast, this work focuses on evaluating an anomaly detection methodology that leverages features from pre-trained networks, also known as backbones, which can be utilized directly without retraining. These backbone networks, trained on large datasets such as ImageNet^37^, have demonstrated the ability to generalize across domains beyond their original training scope^38^. This approach offers a significant advantage by avoiding the computationally expensive and time-consuming process of retraining deep neural networks.

To mitigate the semantic bias of these networks, methods have been engineered to extract features that utilize low-level image neighborhood information. SPADE^39^ builds features through a combination of activations of the first few layers in convolutional neural networks (CNNs) to obtain features for rectangular-sized regions (patches) in the image. Defard et al.^18^ further improved this approach by building a statistical model, a multivariate Gaussian distribution, for the patch features of normal samples and detecting outliers using the Mahalanobis distance. This makes the model size independent of the number of training samples.

We analyze features of pre-trained convolution neural networks^40,41^, which were trained on ImageNet and also a recent self-supervised method based on Vision Transformers^42^, which have shown to provide very good domain adaption capability. All of these methods work on 2D images.

## Method

**Fig. 2:**
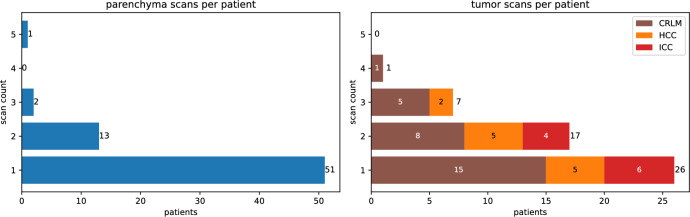
The dataset is comprised of 173 OCT scans taken from 69 patients, labeled as parenchyma or tumor (right) of type CRLM, HCC and iCCA, and contains a varying number of scans per patient, which is visualized in the figure above for parenchyma (left) and tumor (right). The model is trained to differentiate between parenchyma or tumor only.

For the purposes of this study, a pre-existing dataset of 173 OCT scans and corresponding clinical information from 69 patients was used. This data had been collected during previous studies in our institution, according to our published methodology^10,12,13^, with a varying number of scans per patient (Figure 2). Briefly, adult patients undergoing elective oncological liver resections in curative intent at the University Hospital RWTH Aachen (UH-RWTH) between June 2020 and April 2021, who provided informed consent, were included in this study. A table-top spectral domain OCT device (Telesto^TM^ V1, Thorlabs GmbH, Lÿbeck, Germany) was used, operating at 1310 nm wavelength. The field of view was set to 9.90 mm x 2.55 mm x 2.55 mm, with a resolution of 2048 x 512 x 512 pixels and a pixel size of 4.83 $\upmu$m in x-, 4.98 $\upmu$m in y- and 4.97 $\upmu$m in z-direction. Scans were conducted on tumor and parenchyma in fresh resection specimens, in a non-contact technique, leaving some air-tissue interface on the top part of each image. Each volume scan contained 512 B-scans and contained only liver parenchyma or tumor. Scanned areas were marked with pins before placement in formalin and these sections were processed and microscopically examined separately. Additionally, “birds eye view” images of each scan area were captured through the built-in camera of the OCT system. This allowed for high accuracy in matching scans with detailed histological diagnoses. As all scans were either complete tumor or non-tumor-liver-parenchyma, no manual or other annotation of images (or structures within) was carried out. Instead, whole volumes were labelled as “tumor” or “normal” for the purposes of model training and testing. The study was conducted under ethical approval of the Institutional Review Board of the RWTH Aachen University (EK-105/20) and in accordance with the current version of the Declaration of Helsinki, the Declaration of Istanbul, and Good Clinical Practice Guidelines (ICHGCP). All patients provided written informed consent before inclusion in this study.

We formulate the classification task as a semi-supervised problem in which a training set of OCT scans labeled as normal liver parenchyma is used to model the distribution of healthy tissue. Pathological samples are expected to show substantial deviations from this learned distribution. The approach is evaluated on a test set consisting of previously unseen parenchyma scans as well as scans containing confirmed pathological findings.

Prior to applying the anomaly detection method, a pre-processing pipeline is used to extract meaningful 2D image tiles from the 3D scan data. Each scan yields a set of 2D tiles which inherit the histology-derived label assigned to the corresponding scan. The anomaly detection method produces an anomaly score for each tile. To obtain a robust scan-level score, tile-level scores are aggregated per scan using the first quartile, which mitigates the influence of outliers and noise that typically manifest as elevated anomaly scores.

Subsequently, we describe the data preprocessing pipeline and the image-based anomaly-detection method that have been specifically adapted to this application.

### Input data and preprocessing

**Fig. 3:**
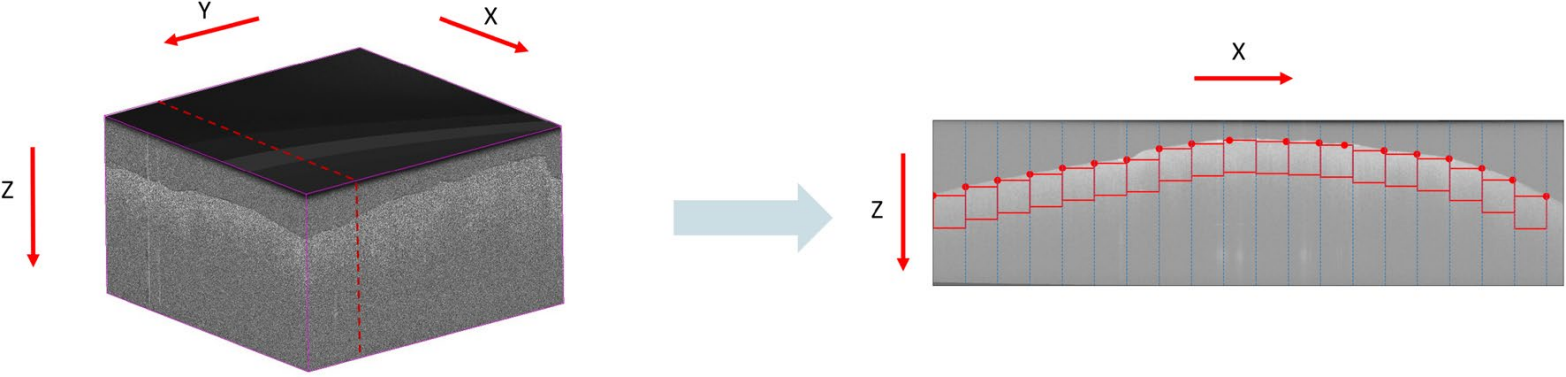
One OCT volume scan volume (left) is sliced into several B-scan images (right), e.g., along the Y axis as shown above. Rectangular tiles are extracted below the tissues surface (red boxes), which are processed by the anomaly detection model.

Each scan, consisting of data from a single tissue specimen, is encapsulated in a separate entity containing additional information such as previews, scan settings, and metadata files. From this entity, we extract two-dimensional B-Scans along both horizontal axes (X and Y) at fixed intervals (Figure 3), preserving the original 12-bit precision and logarithmic intensity scaling. Following an approach similar to that described in^13^, we extract small rectangular tiles below the tissue surface which serve as the context for the machine learning model. After converting the data from 3D to 2D, we also perform an intensity outlier removal to mitigate measurement errors and robust data normalization to help with model stability. A detailed description of the preprocessing steps is provided in the supplementary material.

### Anomaly detection method

**Fig. 4:**
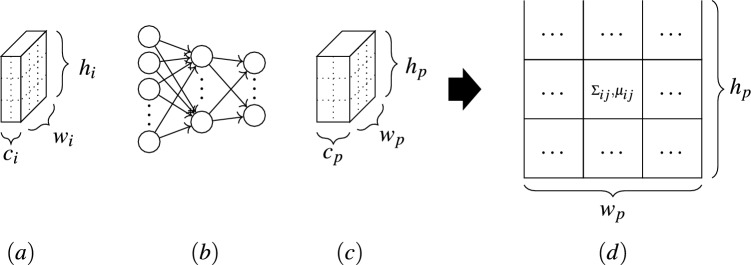
We leverage a patch distribution model (^18^) for anomaly detection: Given an input image (**a**), a tensor of size $(w_i \times h_i)$ pixels (with typically $c_i=3$ input channels), $(w_p \times h_p)$ image patch features *x* with feature channel dimension $c_p$ are extracted (**c**) using a pre-trained backbone network with frozen weights (**b**). A multivariate Gaussian with parameters $\Sigma _{ij}, \mu _{ij}$ is estimated for patch features over the training set (**d**). We evaluate both convolutional and vision transformer backbones in this paper. Given a test sample, an anomaly score is calculated as statistical distance per patch.

We employ a patch distribution modeling framework (PaDiM), as proposed in Defard et al.^18^, for anomaly detection. The general idea is to learn the data distribution of normal data from good examples (parenchyma) and to detect deviations from this distribution as anomalies (tumor). An overview of the method is shown in Figure 4: we adapt a pre-trained backbone network with frozen weights to extract features for small rectangular areas (patches) in the input image. For each patch, we build a statistical model by fitting a multivariate Gaussian (MVG) distribution to the feature vectors of all training samples. This allows us to capture the underlying patterns and variability of parenchyma tissue. Given a test input image, its patch features are extracted and an anomaly score is calculated per patch as a statistical distance to the parenchyma data distribution.

The performance of three backbone networks is analyzed: two convolutional neural network (CNN)-based architectures, specifically ResNet18^40^ and EfficientNetV2^41^, where the MVG is estimated on the CNN feature pyramids as described in the original PaDiM paper. These backbone networks provide features for $4 \times 4$-pixel patches. Additionally, we adapted the CNN-based method to a more recent transformer-based model called DINOv2^42^, which was trained in a self-supervised manner on the LVD-142M dataset^42^. The ViT-S variant of this model is used with registers. We adapt the features of the transformer model by building the MVG on the image patch tokens (features) of the last transformer block, which correspond to patches of $14 \times 14$-pixel size each. The class token is not used. In the training phase, MVG distributions $\mathcal {N}(\Sigma _{ij}, \mu _{ij})$ are empirically estimated from the patch features *x*, a $c_p$-dimensional vector which is the output of the pre-trained network; e.g. $c_p$=384 for the ViT-S used in this paper. Distributions are built for all features of the training set for each patch location *ij*. As detailed in Defard et al.^18^, this means that the patch features only need to be extracted once per sample during training because the feature extractor is pretrained and kept fixed; thus, no deep learning optimization is required.

Given a test sample, patch anomaly scores are calculated using the Mahalanobis distance between a test patch feature $x_{ij}$ and the estimated patch distribution $\mathcal {N}(\Sigma _{ij}, \mu _{ij})$:

1 $$\begin{aligned} M(x_{ij}) = \sqrt{(x_{ij}-\mu _{ij})^T \Sigma _{ij}^{-1} (x_{ij}-\mu _{ij})} \end{aligned}$$

**Fig. 5:**
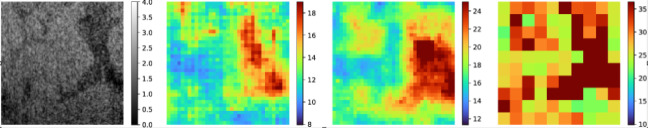
Qualitative comparison of patch-level anomaly scores across different backbone architectures: input tile (far left), ResNet18 (center left), EfficientNetV2 (center right), and DINOv2 (far right). The visualizations reveal that each backbone exhibits distinct patch sizes and varying scales of anomaly scores, highlighting differences in their feature representations and sensitivity to anomalies.

As an example, we show the patch anomaly scores for a selected test sample for the three backbones as visible in Figure 5. Each tile of a B-scan image represents the context window for model evaluation. To classify a scan, we select the maximum patch score within each tile for a per-tile decision, and then aggregate these scores across all tiles to make a per-scan decision.

## Results

We conducted two experiments using a dataset of 173 OCT scans acquired from liver resection samples of 69 patients. Each scan was histologically classified as parenchyma (88 scans) or various tumor types (85 scans of types CRLM, iICCA, and HCC). Since our method is based on anomaly detection, we trained the model exclusively on scans labeled as parenchyma and evaluated its classification performance of parenchyma-versus-tumor on a test set containing both parenchyma and tumor scans of different pathologies. Additionally, because we employ pretrained networks with frozen weights for feature extraction, no separate validation set was used, as the method does not involve network training nor hyperparameter tuning.

In the first experiment, we examined the effect of varying tile sizes and slicing distances on model performance using a smaller subset of 42 scans. Specifically, we used a fixed split of 23 parenchyma scans for training and 19 scans (9 parenchyma, 10 tumor) for testing. Four evaluation datasets were generated by preprocessing the scans with different tile sizes and slicing distances. Each dataset was evaluated using three pretrained backbone networks to identify the optimal configuration.

In the second experiment, we conducted a detailed analysis of the classification behavior distinguishing normal (parenchyma) from anomalous (tumor) tissue. Given the relatively small sample size and the inherent noise in both measurements and annotations, we employed stratified k-fold cross-validation using the best-performing preprocessing and backbone method from the first experiment. We compare different runs by threshold independent metrics and report sensitivity and specificity for a threshold selected to maximize classification accuracy on the balanced test set.

### Evaluation metrics

Given our tile datasets, we train a model using the parenchyma training set data. For testing, we extract patch scores $M_{ij}$ for each test sample (Equation (1)), and as suggested by Defard et al.^18^, we use the maximum of the patch scores as the tile image anomaly score. We chose tumor as the positive label and calculated receiver operating characteristics (ROC) with tile image anomaly scores, along with the tile image classification accuracy after selecting the best-performing threshold.

Using an anomaly score threshold, a predicted image score yields a binary decision label, which is then formulated in a confusion matrix counting true positives (*TP*), false positives (*FP*), false negatives (*FN*), and true negatives (*TN*). Given ground-truth counts for positive *P* and negative *N* test samples, we calculate accuracy $acc=\frac{TP+TN}{P+N}$. The ROC is expressed via the true positive rate $TPR=\frac{TP}{P}$ and the false positive rate $FPR=\frac{FP}{N}$. We utilize the scikit-learn library for metric calculation implementation, and visualize ROC curves plotting *FPR* versus *TPR* and calculate the area under the ROC curve (ROC AUC) for a threshold-independent comparison of experiment runs, we refer to^43^ for more details about ROC analysis.

### Experiment 1: preprocessing strategy and backbone evaluation

We conduct the first experiment on a smaller subset of the available data: 31 parenchyma scans, of which 23 were used for training and 9 for testing, and 10 scans of various tumor types (5 CRLM, 3 iCCA, 1 CCC, 1 prostate CA metastasis).

Four training datasets are created using varying sampling distances between cross-sections (B-Scans) and different tile sizes, resulting in varying numbers of B-Scans and tiles (Table 1).

**Table 1:** Training and test data sizes for various B-scan sampling distance and tile size, obtained from processing 31 parenchyma C-scans and 10 tumor C-scans. As the anomaly detection model operates on tile level, train and test tiles have been split by C-scans, such that the test tiles classes contain a similar number of tiles.

Id	Sampling	B-scans	Tile size		Train tiles	Test tiles	
	[mm]		[px]	[mm]	Parenchyma	Parenchyma	Tumor
v1	0.5	552	126	0.62	3,288	1,269	1,397
v2	0.5	552	252	1.24	1,427	547	600
v3	0.25	1,127	252	1.24	2,883	1,107	1,209
v4	0.1	2,829	140	0.69	14,564	5,669	6,352

Data sets v1 to v4 are used to assess the parenchyma-versus-tumor classification accuracy on the OCT-data of three backbones: ResNet18 and EfficientNetV2-S with the official pre-trained ImageNet weights available from torchvision, and DINOv2 with official VIT-S/14 weights pre-trained on LVD-142M. We also test the effect of reducing patch feature dimension size. Results generated with different backbone embedding sizes and a number of tiles in the training dataset are compared to evaluate their influence on the tile classification accuracy. In the process individual tiles are classified as parenchyma (normal) and tumor (anomaly) according to their respective anomaly score based on a separation threshold. The results show clear advantages for the Vision Transformer-based approach with DINOv2 for all four datasets:

**Fig. 6:**
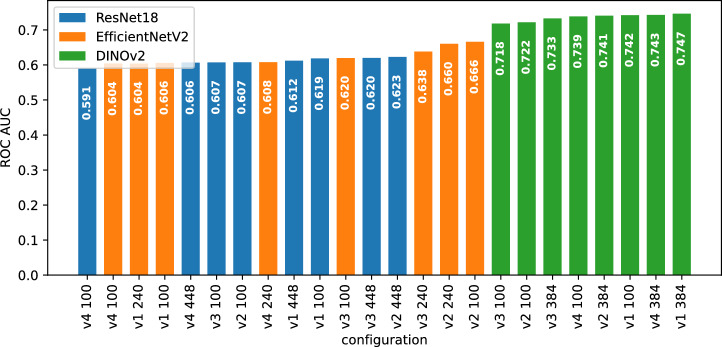
Experiment 1: Evaluation of backbone performance under varying tile sizes, slicing distances (as indicated by dataset versions), and embedding dimensions. Models are compared using tile-level classification accuracy of parenchyma versus tumor. The Vision Transformer-based DINOv2 achieves the highest 2D tile classification performance.

The accuracy scores presented in Figure 6 indicate that the choice of the backbone architecture has a notable impact on classification accuracy. In contrast, the number and size of tiles used during training, as well as the embedding size, have a comparatively minor effect on the accuracy scores. Specifically, for the Vision Transformer-based backbone DINOv2, the variation in accuracy score is smaller. We chose strategy v4 as it is in the best performing range with a small tile size (140x140 pixel).

### Experiment 2: k-fold cross validation

Using the best performing model of experiment 1 (Tile sampling strategy v4 with DINOv2 backbone with 384-dimensional patch features), we further analyze the parenchyma-versus-tumor classification performance using a larger dataset.

We included 88 parenchyma scans and 85 tumor scans (50 CRLM, 14 iCCA, and 21 HCC), confirmed by histology. While globally balanced, the tumor subtypes were imbalanced, and the number of scans per patient varied between 1 and 5, as shown in Figure 2. To account for known noise in the data, we employed a scan-wise stratified k-fold cross-validation strategy with $k=4$, as the detection task focuses on local intraoperative margins rather than patient-level outcomes. This involved splitting the 88 parenchyma scans into training (66 scans) and testing (22 scans) sets. We ensured a balanced representation of tumor types (CRLM, iCCA, and HCC) in both the training and testing sets to maintain the same size as the parenchyma testset across all splits, the exact train and testset sizes are shown in Table 2.

**Table 2:** Summary of the 4-fold stratified cross-validation split for parenchyma-vs-tumor classification. C-scan counts are provided for both training and test sets (parenchyma and tumor subtypes), with balanced class representation across folds. Tile-level sample sizes are also reported for each fold.

Run	*Train*		*Test*			
	Parenchyma		Parenchyma		Tumor (CRLM + iCCA + HCC)	
	C-scans	tiles	C-scans	tiles	C-scans	tiles
1	66	40,897	22	13,791	21 (12 + 4 + 5)	13,120
2	66	41,036	22	13,652	21 (13 + 3 + 5)	13,310
3	66	41,016	22	13,672	21 (12 + 4 + 5)	13,086
4	66	41,115	22	13,573	22 (13 + 3 + 6)	13,834

**Fig. 7:**
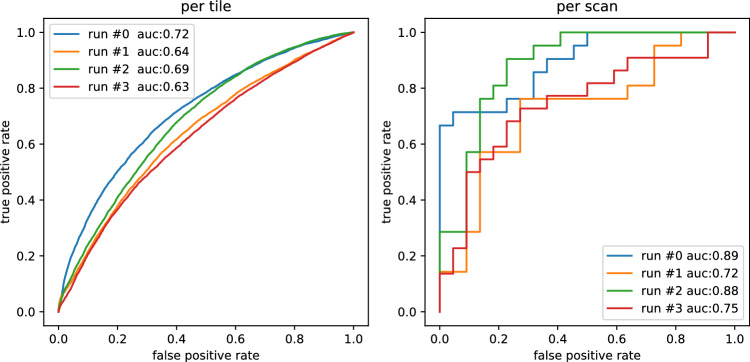
We show receiver-operator characteristic (ROC) curves for the parenchyma-versus-tumor classification task (with tumor defined as the positive label) using a four-fold cross validation scheme. In each fold, the data are split into a parenchyma-only training set and a test set containing samples of both parenchyma and various tumors. The left ROC curves illustrates classification performance at the tile level, whereas the right ROC curves shows scan-level performance, obtained by aggregating the tile-level scores using the first quartile. The improved scan-level performance indicates that this aggregation strategy effectively mitigates the impact of noise and outliers (mean improvement: 14.3%).

As shown in Table 3 and Figure 7, our experiments achieved a mean ROC AUC of 0.668 per tile level, corresponding to a mean classification accuracy of $62.7\%$ (using the separation threshold that maximizes accuracy on the balanced test set for each fold). We observed that scans may contain noise or outlier tiles, thus, we note that this mediocre result can be improved by grouping the information per scan and making the classification decision per scan volume. We group tile scores per scan, and as outlier and noise corresponds to higher anomaly scores, we choose the first quartile of the grouped tile scores as the score per scan. Using these scores, we evaluate the classification performance of the test set as AUC for each fold, and calculate a p-value indicating statistical significance against random chance (null hypothesis AUC=0.5). As an aggregated metric over all folds, we use out-of-fold pooling and stratified bootstrapping to calculate a pooled AUC of 0.816 (95% CI 0.748-0-875); the pooled p-value for a Mann-Whitney U test (null hypothesis AUC=0.5) was <0.001.

For a better interpretability, we also calculate threshold-dependent metrics sensitivity and specificity in Table 3 by choosing the best-separating threshold on the test set, according to the classification accuracy (we refer to Evaluation Metrics for accuracy calculation).

**Table 3:** Summary of a stratified 4-fold evaluation for parenchyma-vs-tumor classification using the aggregated C-scan anomaly score. ROC AUC is reported as the primary threshold-independent metric, with p-values indicating statistical significance against random chance (null hypothesis AUC = 0.5). Mean class anomaly scores, the accuracy-maximizing threshold on the balanced test set folds, and corresponding sensitivity and specificity are also reported. Aggregating out-of-fold test scores across all folds, the pooled AUC was 0.816 (95% CI: 0.748–0.875; pooled p-value < 0.001).

Fold	ROC AUC	p-value	Parenchyma	Tumor	Score			
			Score mean	Score mean	Threshold	Accuracy	Sensitivity	Specificity
1	0.894	<0.001	21.03	25.91	22.78	0.837	0.71	0.95
2	0.722	0.006	22.17	25.03	22.81	0.744	0.76	0.73
3	0.883	$<0.001$	21.91	25.48	22.65	0.837	0.90	0.77
4	0.746	0.002	22.07	24.33	22.30	0.727	0.73	0.73
Mean	0.811		21.80	25.19	22.63	0.79	0.78	0.8

Our analysis revealed noise present in the data. The patch anomaly score, reflecting the degree of non-normality, offers valuable insights into the model’s behavior. A detailed analysis of grouped tile scores for each scan across all four cross-validation runs is provided in the supplementary material.

**Fig. 8:**
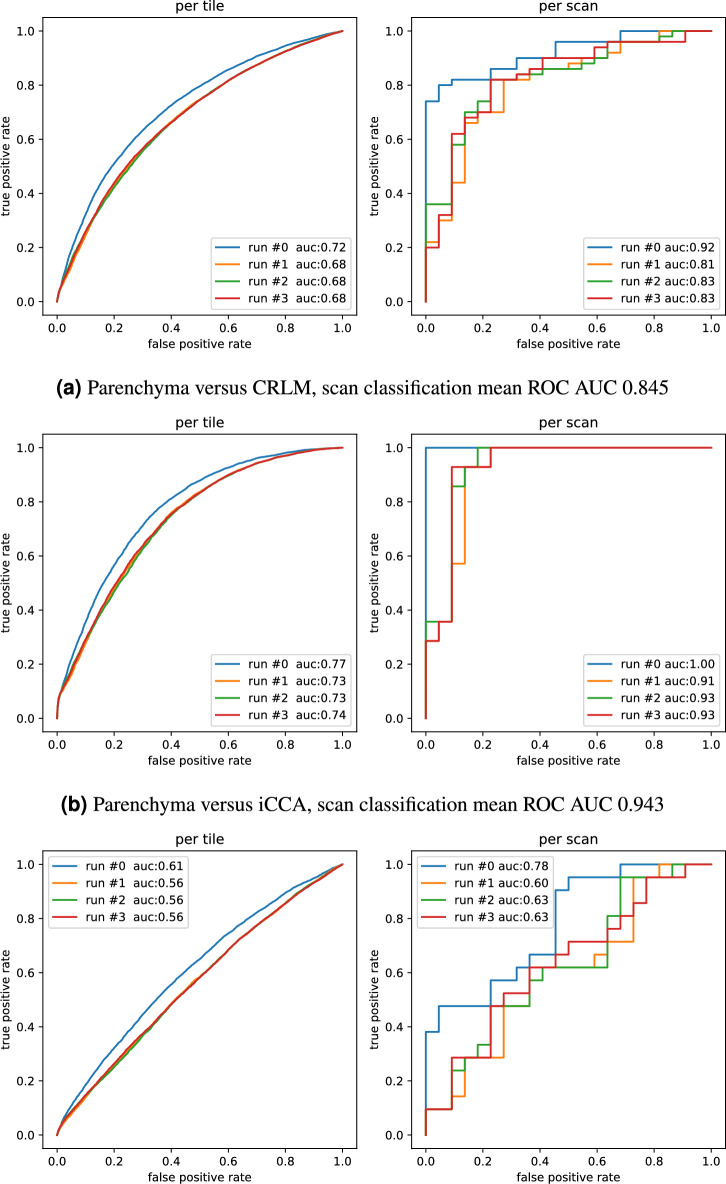
We further evaluate parenchyma-versus-tumor classification performance at the tile level (left) and the scan level (right) using an alternative data split in which the parenchyma subset remains unchanged, but only pathological samples from a single tumor type are included. The results demonstrate that classification performance varies across tumor types, with iCCA and CRLM exhibiting strong performance, whereas HCC shows comparatively weaker performance.

**Per-Tumor Analysis**: We also performed an analysis per tumor type (Figure 8): due to the relatively low number of tumor samples (50 CRLM, 14 iCCA, and 21 HCC), we keep the training splits of parenchyma scans and always evaluate against all tumor samples of a specific class for each fold.

**Fig. 9:**
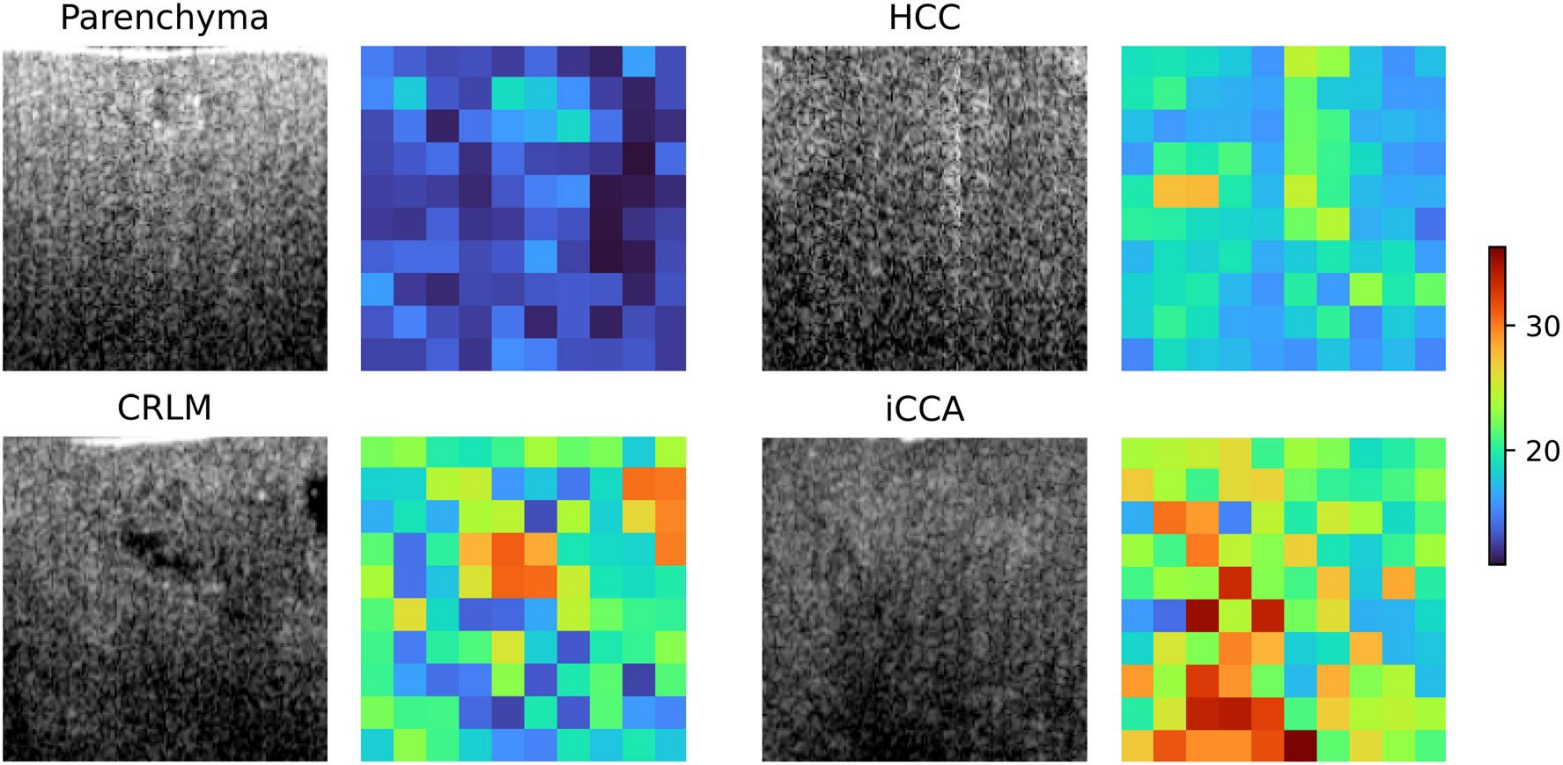
A qualitative comparison of model inputs and outputs (patch-level scores) at the tile level is shown. The model input size is 140x140 pixels, anomaly scores, are calculated for 10x10 patches, displayed to the right of the corresponding input tiles. A blue color represents a low statistical distance to normal (parenchyma) tissue, and red a high distance (anomaly). The anomaly scores indicate higher values for tiles extracted from tumor scans, with scores increasing progressively for HCC, CRLM, and iCCA. All anomaly maps are visualized using a consistent color scale to ensure comparability.

Our analysis revealed distinct tumor-specific classification behaviors (Figure 8). iCCA demonstrated superior recognition performance, followed by CRLM with comparatively good performance. Notably, HCC exhibited poor performance. Furthermore, Run 0 consistently achieved the highest accuracy, suggesting the presence of noise within our training dataset. An example of a qualitative comparison of anomaly patch scores for parenchyma and various tumor types is presented in Figure 9.

### Runtime characteristics

All experiments were run using an Intel Xeon 6346 3.10Ghz compute server with an Nvidia A40 GPU (single GPU instance only). The maximum run time for one k-fold run of experiment 2 is 86 seconds for 40k training tiles (66 parenchyma scans). Testset inference time for 26k parenchyma and tumor tiles is 33 seconds in total.

The model consists of 36.8M parameters for the pretrained vision transformer backbone (DINOv2 ViT-S) and 14.7M patch distribution parameters.

## Discussion

This study demonstrates that OCT combined with anomaly detection can accurately differentiate between malignancies (HCC, iCCA, CRLM) and normal liver parenchyma ex vivo. Given the limited dataset size, we evaluated tumor detection performance using a 4-fold stratified cross-validation strategy. The anomaly detection model was trained on 75% of the available parenchyma scans, while the test set comprised the remaining 25% of parenchyma scans and a stratified split of tumor scans for each fold. We evaluated threshold-independent classification by out-of-fold pooling with ROC AUC 0.816 (95% CI 0.748-0.875). For better interpretability we also calculate a threshold for each fold that maximizes classification accuracy on the test set, where the mean sensitivity, specificity, and accuracy were 0.78, 0.8, and 0.79, when considering partial two-dimensional scans extracted from below an organ’s surface. The mean C-scan anomaly score for parenchyma was determined to be 21.8, for tumor 25.19 with a mean decision threshold at 22.63.

In our previous work, we combined OCT and convolutional neural networks (CNN) to distinguish CRLM^12^ and iCCA^13^ from liver parenchyma ex vivo. Those were among the few studies on OCT and liver in the literature. Apart from ex vivo animal studies on OCT technical properties^44,45^, or grading of steatosis, inflammation and^46,47^, there have been limited studies in human livers. These include proof-of-concept studies on formalin-fixed or in vitro liver tissues^48–50^ and one diagnostic study using a support vector machine (SVM) model to distinguish HCC from healthy liver parenchyma^51^.

To the best of our knowledge, this study is the first one to apply anomaly detection to OCT images of human liver tissues. Although carried out on OCT images collected in our previous work^12,13^, the applied methodology of image analysis and model training was novel. We specifically employed a statistical approach to model outliers by leveraging feature distributions from good samples only estimated using pre-trained neural networks^18^. Our evaluation of different backbone architectures revealed the effectiveness of a recent Vision-Transformer-based pre-trained network (DINOv2)^42^ over convolutional alternatives^40,41^. Beyond aggregate performance, the transformer backbone is particularly well-suited to our anomaly-detection setup (PaDiM): its tokenization and global self-attention yield semantically richer patch embeddings, which in turn produce better-conditioned multivariate Gaussian estimates at patch locations. In our pipeline, frozen multi-layer features from DINOv2 provide complementary scales of representation that PaDiM models efficiently, improving the statistical separation between parenchyma and tumor tiles. To our knowledge within this setting (OCT of liver specimens), this ViT-PaDiM pairing has not been systematically evaluated, and our results indicate that the combination is advantageous both at the tile level and after scan-level aggregation. In contrast to traditional supervised learning, our approach offers two key advantages: it skips the computationally expensive step of training neural network weights, thus saving time and computational resources, and it also reduces the need for large amounts of labeled training data by relying on good samples only. Furthermore, the method produces an anomaly score - a quantitative measure that indicates the severity of anomalies at each location in the image - providing valuable insights into the results and significantly enhancing their interpretability. However, the score is tied to the training dataset and backbone used for feature extraction, which means anomaly scores will be different when these factors change.

The inclusion of different tumor types and the training on normal liver parenchyma are significant: clinically, it is often enough to detect malignancy at resection margins, peritoneal lesions, or lymph nodes without a specific histological diagnosis. The decision to extend a liver resection until margins are clear or to end an operation and pursue systemic therapy can be taken there and then. Therefore, the ability of the described methodology to reliably and accurately distinguish malignant from normal liver tissue is a promising first step on which further work can be carried out. The ability to train on normal tissue alone is advantageous, as acquiring normal liver tissue (both in vivo and ex vivo) is significantly easier than obtaining tumor tissue.

Our study demonstrated the potential for the incorporation of OCT and anomaly detection in future in vivo clinical applications. As discussed, these could include intraoperative examination of liver resection margins, peritoneal lesions, or lymph nodes. This would reduce the number of frozen sections and total operation time^52^. Additionally, it could be used for the earlier and more accurate diagnosis of iCCA during endoscopic retrograde cholangiopancreatography (ERCP). Concomitant use of OCT and biliary brushing improves diagnostic sensitivity^53^ and the addition of anomaly detection could further improve on this. Especially relevant questions are the distinction between malignancy and pre-malignant conditions from inflammation, as well as the accurate detection of tumors in scans, which only partially contain them. For these applications, a large dataset encompassing high-quality OCT data from tumors, inflammation, and healthy tissue must be acquired.

The relatively weak performance of our algorithm in distinguishing liver parenchyma from HCC, compared with the classifications of liver parenchyma versus iCCA or versus CRLM, is likely attributable to several factors. First, because HCC arises from hepatocytes, its morphology can closely resemble that of the surrounding non-tumorous liver tissue. This similarity is particularly pronounced in well-differentiated HCC^54,55^, making discrimination more challenging. In contrast, iCCA and CRLM originate from non-hepatocytic cell lineages and display architectural and histopathological patterns that are more distinct from normal liver parenchyma^54^, facilitating their separation by a convolutional neural network. Additionally, HCC is characterized by substantial molecular and histological heterogeneity^55^. When only limited training data are available, this variability may prevent the model from capturing the full spectrum of features required for reliable classification. To further enhance our anomaly detection model, potential improvements could include integrating newer foundation models that have been specifically designed for image processing tasks or adapting a backbone to the OCT-specific domain to fine-tune features that are better suited for anomaly detection than those from pre-trained networks trained on natural images. However, such enhancements would require an additional training step.

Finally, as previously discussed^12^, OCT devices with different wavelengths and polarization-sensitivity should be employed, to potentially increase diagnostic capability and accuracy in different situations. For example, a shorter wavelength (with higher resolution) could be used to scan bile ducts. Polarization-sensitive OCT has been shown to effectively display fibrotic tissue and microvascular complexes in murine livers^47^. This should be the aim of further studies, starting ex vivo and expanding into the in vivo domain. Certain limitations of the present study must be considered when interpreting our results. While the method relies on statistical modeling using frozen backbone weights to detect deviations from the parenchyma distribution rather than memorizing specific tumor features via weight optimization, the small sample size (N=173) limits generalizability and introduces a potential risk of overfitting. As a proof-of-concept study, our primary evaluation focuses on threshold-independent ROC AUC metrics to compare backbone architectures. Future work with a larger, independent validation cohort will be required to establish a clinically validated decision threshold and enable external validation to validate the generalization of the approach. However, as previously mentioned, there are no comparable studies in the literature. Consequently, this study serves as a proof of concept and establishes a baseline, on which further work can be based. Furthermore, an upgraded OCT system that is able to provide quicker and higher-quality scanning without delaying the histological processing of tissues would certainly help. The system used in this study is relatively old and suffers from limitations in memory and processing power, which restricted the quality of scans (e.g., resolution, noise filtering, scan dimensions), as well as the number of scans possible per resection specimen. Additionally, this study focused on liver parenchyma vs. tumor, whereas in real clinical settings, other pathologies must be considered, such as inflammation and pre-malignant stages. Especially for clinical questions concerning lymph nodes or the peritoneum, different control tissues are required.

To address these limitations and build on this work, further studies are needed, starting ex vivo and expanding into the in vivo domain. Future work should focus on improvements in scanning methodology, acquisition of larger data volumes from a variety of tissues and pathologies, as well as more detailed labeling of images for training of deep-learning algorithms. For this, prospective studies are necessary, ideally in a multicentric setting, with more detailed clinical and histopathological information (e.g., information on chemotherapy and tumor regression, detailed reporting on liver parenchyma including steatosis, fibrosis, inflammation, expansion to tissues such as pancreas, bile ducts, or peritoneal lesions). A particularly interesting addition to the OCT system would be polarization sensitivity, as this has been shown to effectively display fibrotic tissue and microvascular complexes in murine livers^47^.

The datasets generated and/or analyzed during the current study are available from the corresponding author on reasonable request.

## Conclusion

Optical coherence tomography combined with anomaly detection enables reliable differentiation between malignant liver lesions (HCC, iCCA, CRLM) and non-neoplastic liver parenchyma in the ex vivo setting. This approach demonstrates strong potential for the development of rapid and accurate intraoperative diagnostic tools to assess resection margins and characterize suspicious hepatic lesions. Further investigations are warranted to validate and refine these findings, including the integration of additional tissue entities and heterogeneous tissue compositions that more closely reflect real-world clinical senarios.

## Data Availability

Data used and/or generated in this study are available upon reasonable request to the corresponding author.
